# Clinical Efficacy of Scaling and Root Planing With Placental Extract Gel Under Magnification in Chronic Periodontitis Patients: A Split-Mouth Study

**DOI:** 10.7759/cureus.61851

**Published:** 2024-06-06

**Authors:** Surabhi Bhadauriya, Sanjay Vasudevan, Ajay Reddy Palle, Abhinav Atchuta, Anuradha Singh

**Affiliations:** 1 Periodontology, Army College of Dental Sciences, Secunderabad, IND

**Keywords:** chronic periodontitis, non-invasive periodontal therapy, placentrex, local drug delivery, placental extract gel

## Abstract

Background

Chronic localized periodontitis is a prevalent and persistent inflammatory condition in which there is the gradual degradation of the gingiva, periodontal ligament fibers, and alveolar bone loss. The objectives of periodontal therapy encompass not solely the elimination of local factors from the periodontal pocket but also the eradication of the dysbiotic microbial milieu to restore periodontal health. The present study aimed to compare the efficacy of scaling and root planing (SRP) with and without the placement of placental extract gel in the therapeutic management of chronic localized periodontitis under magnification.

Materials and methods

The present investigation encompassed 40 sites in 20 systemically healthy patients with chronic localized periodontitis. The allocation of the sites was done randomly, resulting in two distinct groups: group I (test site) and group II (control site). Group I was subjected to SRP, followed by the placement of placental extract gel, while group II solely received SRP. Clinical evaluations of pocket probing depth, plaque index, relative attachment level (RAL), gingival index (GI), and bleeding on probing (BoP) were performed at each site at baseline, six weeks, and three months.

Results

Placental extract gel as an accompaniment to SRP showed significant improvement in clinical parameters like pocket probing depth, RAL, GI, and BoP.

Conclusion

Placental extract gel may significantly act as a local drug delivery agent in the treatment of localized periodontal pockets.

## Introduction

Chronic periodontitis is characterized clinically by gingival tissue attachment loss to the tooth, widening of the gingival crevice (hereinafter “periodontal pocket” in periodontitis), periodontal ligament deterioration, and alveolar bone loss [1]. This destructive mechanism is linked to developing subgingival microbial populations and a dense immuno-inflammatory infiltration in the periodontium, which can lead to tooth loss if not treated properly.

Periodontal therapy intends to alter or remove the pathological microbial environment and risk factors for periodontitis to comprehend the disease progression and restore dentition in health and function with appropriate esthetics [2]. Typically, phase I therapy and surgical root debridement do not effectively remove periodontal pathogens like *Porphyromonas gingivalis*, *Peptostreptococcus micros*, *Prevotella intermedia*, enteric rods, *Bacteroides forsythus*, *Aggregatibacter actinomycetemcomitans*, and possibly other pathological microbes from the subgingival environment [3,4]. These periodontal pathogens are highly invasive, penetrating the gingival epithelium and connective tissues, and they have a strong affinity for the dentinal tubules and sulcular epithelium. This makes it difficult to completely remove these microbes through surgical debridement of periodontal tissues [1,5].

The administration of systemic and local antibiotic agents in periodontal pockets may inhibit the growth of periodontal pathogens, enhancing the effects of conventional phase I periodontal therapy. Hence, it is important to devise better strategies for delivering medications with a controlled approach directly into the periodontal pocket to be treated, reducing unwanted effects such as systemic absorption of antibiotics and bacterial resistance [6].

Derived from traditional insights, human placental extract has been utilized for therapies such as wound healing, ophthalmic conditions, infertility, stroke, epilepsy, and others. It is a copious source of regenerative agents, such as proteins, biological catalysts, hormones, mucopolysaccharides, polynucleotides, and so on, and therefore, the evolution of such a multifunctional and vital organ is seen as a rational and advantageous outcome of natural selection, optimizing reproductive success and well-being of the fetus [7]. Within the body, an aqueous extract of the human placenta increases the metabolic activity in cells of the periodontium, which provides energy to respond to periodontal inflammation. It also assists in the removal of granulation and infected tissue, preventing exudate formation and managing the bacterial load. Aqueous placental extract includes nucleotides like polydeoxyribonucleotides and nicotinamide adenine dinucleotide phosphate hydrogen, which are known for their regenerative properties. Additionally, it supplies growth factors, amino acids, and small peptides that support matrix formation and cell adhesion, enhancing wound healing [8,9]. Furthermore, nitrous oxide induction and antibacterial activity of the extract against several pathogenic and drug-resistant strains have been demonstrated, implying at least partial protection against subsequent infections in chronic wounds [10,11]. With such therapeutic properties, the use of human placental extract as a local drug delivery along with scaling and root planing (SRP) could become a promising novel therapy in the treatment of chronic periodontitis. 

Research has also demonstrated that magnification loupes improve the precision of instrumentation and enhance the visualization of the oral cavity [12]. This study sought to combine the therapeutic benefits of placental extract gel with a microsurgical approach using dental loupes during the initial phase of periodontal disease treatment in order to develop an innovative therapy for chronic periodontitis.

The present study aimed to compare the clinical soft tissue parameters, i.e., gingival index (GI), plaque index (PI), bleeding on probing (BoP), periodontal probing depth (PPD), and relative attachment level (RAL), recorded in chronic periodontitis patients treated with SRP with and without placement of placental extract gel as a local drug delivery under magnification.

## Materials and methods

This investigation included the patients visiting the Department of Periodontics in the institution. The study was approved by the institutional ethical committee and was registered at ctri.nic.in with CTRI no. CTRI/2023/04/051521. As per the Declaration of Helsinki and good clinical practice guidelines, all guidelines were followed.

The study enrolled 40 sites in 20 participants with chronic localized periodontitis. The inclusion criteria consisted of individuals aged 20-45 years who had localized PPD of 4-6 mm. The exclusion criteria encompassed individuals with systemic diseases that impact periodontal health, pregnant or lactating women, smokers, alcoholics, and tobacco consumers. Patients with a history of periodontal therapy in the past six months were also excluded. The study design was split-mouth, in which the selected sites in each individual were divided randomly by the “allocation concealment” method into two groups: group I (test group) and group II (control group).

**Figure 1:**
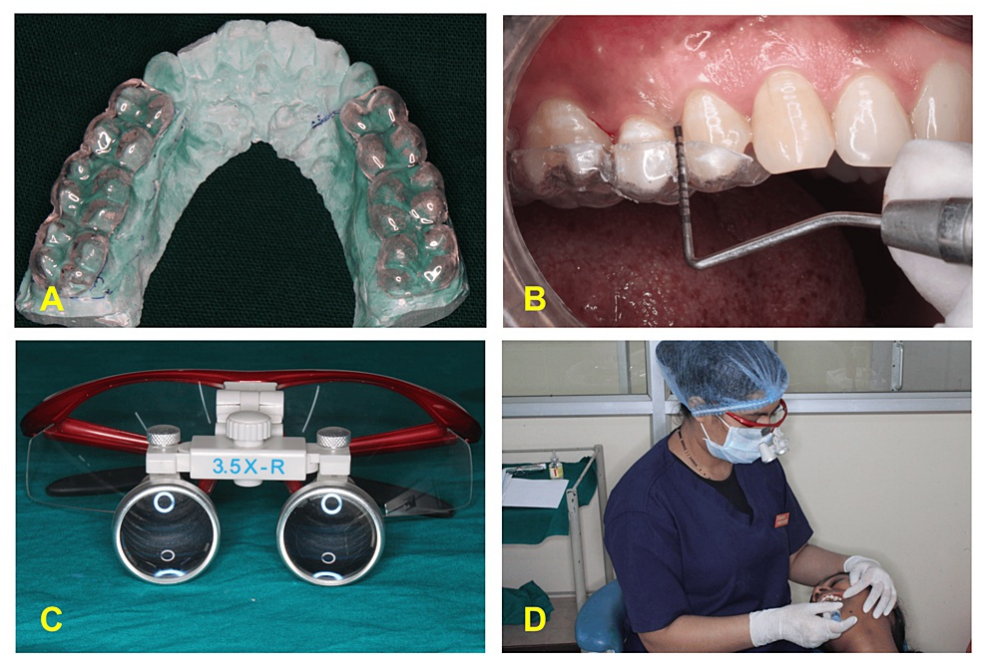
Recording of the clinical parameters (A) Acrylic stent prepared on the diagnostic model to record RAL. (B) Placement of stent at the site and recording of RAL and pocket depth using UNC-15 probe. (C) Magnification loupes. (D) Operator recording the parameters while wearing magnification loupes. RAL, relative attachment level; UNC-15 probe, University of North Carolina

After the selection of sites according to inclusion and exclusion criteria, impressions were made using alginate impression material. An acrylic stent was made on the models to record RAL (Figure 1A). Clinical parameters were recorded at baseline (Figure 1B).

The patients were assessed for the following clinical parameters: GI (Loe and Silness, 1963), PI (Silness and Loe, 1964), BoP, PPD, and RAL [13]. A single investigator assessed these parameters using a University of North Carolina-15 (UNC-15) periodontal probe, with the entire procedure conducted using magnification loupes at 3.5× magnification (Figures 1C, 1D).

**Figure 2:**
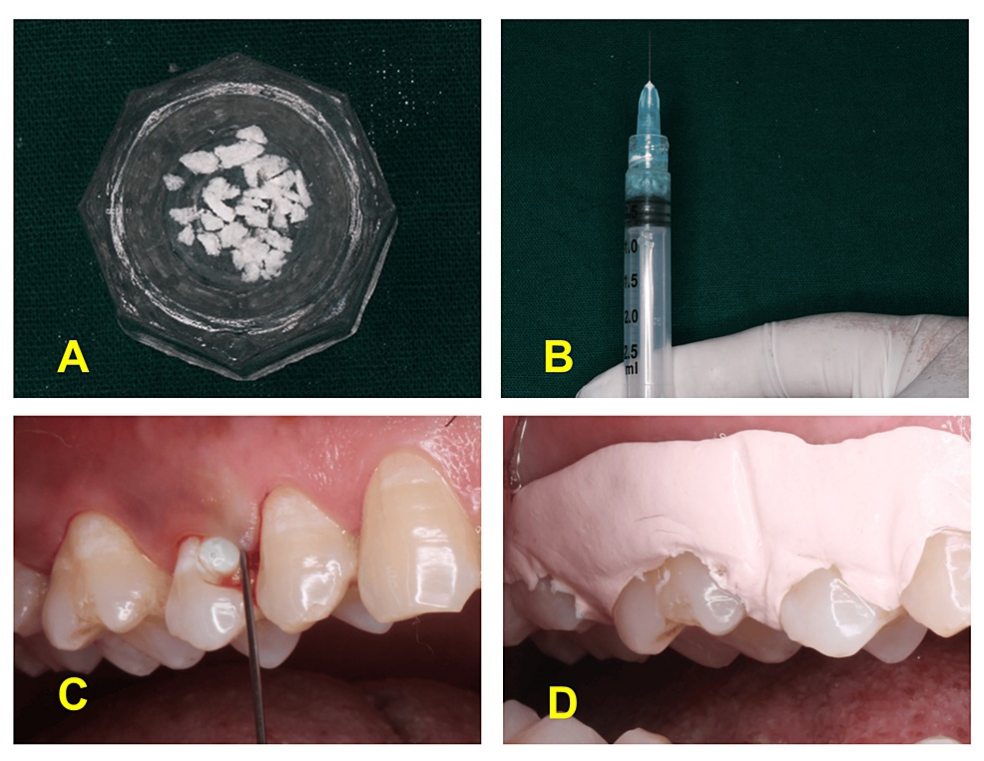
Placement of placentrex (A) Collagen plug. (B) Mixing of collagen plug with the placental extract gel. (C) Placement of placental extract gel till the pocket is filled completely. (D) Coe Pak placement.

Subsequently, the sites in group I were treated with SRP using an ultrasonic scaler, followed by the placement of 1 mL of placental extract gel (Placentrex® Gel, Albert David) adsorbed in a resorbable collagen plug (Fix Plug™, Synerheal) (Figures 2A-2C) and the placement of Coe Pak after completion of the procedure (Figure 2D), while sites of group II underwent only SRP with an ultrasonic scaler. The treatment for both groups was performed using 3.5× magnification.

The first follow-up was done on the seventh day to remove the Coe Pak from test sites without intervening in the treated area. A second follow-up was done after six weeks, and a re-evaluation of the clinical parameters was recorded. The third follow-up was done after three months for the final clinical evaluation.

Statistical analysis was carried out using IBM SPSS Statistics, version 23.0 (IBM Corp., Armonk, NY, USA). The data interpretation was done with the mean and standard deviation. To compare within groups, statistical analysis methods such as the Friedman test, repeated measures ANOVA test, and Cochran’s Q test were employed. The intergroup comparison was also conducted using the independent t-test, chi-square test, and Mann-Whitney test.

## Results

In the present study, it was observed that placental extract gel was well tolerated by all subjects, with no reported adverse effects such as allergy, burning sensation, swelling, or discoloration, among others. Furthermore, intragroup and intergroup comparisons were made for the clinical parameters.

In intragroup comparison, there was a significant improvement in GI, PI, and RAL scores in both test and control groups (Tables 1, 2, respectively) from baseline to six weeks and three months (p-value < 0.001), but the improvement was not significant from six weeks to three months.

**Table 1:** Intragroup comparison of clinical parameters (group I-test) *A significant difference at p ≤ 0.05 (statistically significant) SD, standard deviation

Parameter	Interval	Mean ± SD	p-value
Gingival index (GI)	Baseline	1.75 ± 0.47	Baseline vs 6 weeks: <0.001*
	6 weeks	0.83 ± 0.43	Baseline vs 3 months: <0.001*
	3 months	0.66 ± 0.47	6 weeks vs 3 months: 0.707
Plaque index (PI)	Baseline	1.43 ± 0.61	Baseline vs 6 weeks: <0.001*
	6 weeks	0.66 ± 0.55	Baseline vs 3 months: <0.001*
	3 months	0.55 ± 0.50	6 weeks vs 3 months: 0.707
Periodontal probing depth (PPD) (mm)	Baseline	4.65 ± 0.67	Baseline vs 6 weeks: <0.001*
	6 weeks	2.90 ± 0.91	Baseline vs 3 months: <0.001*
	3 months	2.65 ± 0.67	6 weeks vs 3 months: 1.000
Relative attachment level (RAL) (mm)	Baseline	10.70 ± 1.17	Baseline vs 6 weeks: <0.001*
	6 weeks	8.85 ± 1.35	Baseline vs 3 months: <0.001*
	3 months	8.70 ± 1.38	6 weeks vs 3 months: 0.248

**Table 2:** Intragroup comparison of clinical parameters (group II-control) *A significant difference at p ≤ 0.05 (statistically significant) SD, standard deviation

Parameter	Interval	Mean ± SD	p-value
Gingival index (GI)	Baseline	1.64 ± 0.46	Baseline vs 6 weeks: <0.001*
	6 weeks	1.04 ± 0.52	Baseline vs 3 months: <0.001*
	3 months	0.99 ± 0.54	6 weeks vs 3 months: 1.000
Plaque index (PI)	Baseline	1.28 ± 0.57	Baseline vs 6 weeks: <0.001*
	6 weeks	0.60 ± 0.45	Baseline vs 3 months: <0.001*
	3 months	0.53 ± 0.50	6 weeks vs 3 months: 1.000
Periodontal probing depth (PPD) (mm)	Baseline	4.40 ± 0.50	Baseline vs 6 weeks: 0.003*
	6 weeks	3.70 ± 0.66	Baseline vs 3 months: 0.003*
	3 months	3.70 ± 0.66	6 weeks vs 3 months: 1.000
Relative attachment level (RAL) (mm)	Baseline	10.15 ± 1.57	Baseline vs 6 weeks: <0.001*
	6 weeks	9.45 ± 1.50	Baseline vs 3 months: <0.001*
	3 months	9.45 ± 1.50	6 weeks vs 3 months: 1.000

The intragroup comparison of the change in PPD in group I and group II (Tables 1, 2, respectively) showed that the PPD reduced significantly from baseline to six weeks (p-values of <0.001 and 0.003, respectively) and baseline to three months (p-values of <0.001 and 0.003, respectively) in both groups, but the changes were insignificant from six weeks to three months. Also, intergroup comparison (Table 3) revealed that the GI scores of the two groups did not differ at baseline. However, the GI score of group I was significantly lower than that of group II at six weeks (p-value = 0.041) and three months (p-value = 0.007).

**Table 3:** Intergroup comparison of clinical parameters *A significant difference at p ≤ 0.05 (statistically significant) SD, standard deviation

Parameter	Interval	Group I (test)	Group II (control)	Difference	p-value
		Mean ± SD	Mean ± SD		
Gingival index (GI)	Baseline	1.75 ± 0.47	1.64 ± 0.46	0.11	0.330
	6 weeks	0.83 ± 0.43	1.04 ± 0.52	-0.21	0.041*
	3 months	0.66 ± 0.47	0.99 ± 0.54	-0.33	0.007*
Plaque index (PI)	Baseline	1.43 ± 0.61	1.28 ± 0.57	0.15	0.541
	6 weeks	0.66 ± 0.55	0.60 ± 0.45	0.06	0.604
	3 months	0.55 ± 0.50	0.53 ± 0.50	0.02	0.776
Periodontal probing depth (PPD) (mm)	Baseline	4.65 ± 0.67	4.40 ± 0.50	0.25	0.244
	6 weeks	2.90 ± 0.91	3.70 ± 0.66	-0.80	0.002*
	3 months	2.65 ± 0.67	3.70 ± 0.66	-1.05	<0.001*
Relative attachment level (RAL) (mm)	Baseline	10.70 ±1.17	10.15 ± 1.57	0.55	0.216
	6 weeks	8.85 ± 1.35	9.45 ± 1.50	-0.60	0.192
	3 months	8.70 ± 1.38	9.45 ± 1.50	-0.75	0.109

**Figure 3:**
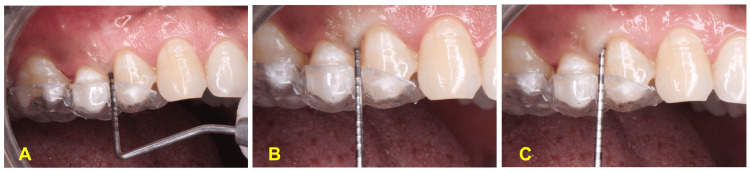
Pocket probing depth (A) Baseline. (B) Six-week follow-up. (C) Three-month follow-up.

The PI scores and RAL of the two groups (Table 3) did not show any significant difference at baseline, six weeks, and three months. The intergroup comparisons of PPD (Table 3) showed that the test group had significantly reduced PPD at six weeks and three months (Figures 3A-3C) compared to the control group (p-values of 0.002 and <0.001, respectively). The intergroup comparison did not show any significant difference in RAL in both groups.

The BoP was present in all the sites at baseline in both groups. In group I, the BoP was present only at three sites after six weeks, while in group II, it was present at 10 sites. Also, after three months, BoP was absent at all the sites in group I, while nine sites in group II were still present with BoP (Tables 4, 5).

**Table 4:** Intragroup comparison of bleeding on probing *A significant difference at p ≤ 0.05 (statistically significant) SD, standard deviation

Group	Interval	Yes	No	p-value	Pairwise comparisons
Group I (test)	Baseline	20 (100%)	0	<0.001*	Baseline vs 6 weeks: <0.001*
	6 weeks	3 (15%)	17 (85%)		Baseline vs 3 months: <0.001*
	3 months	0	20 (100%)		6 weeks vs 3 months: 1.000
Group II (control)	Baseline	20 (100%)	0	<0.001*	Baseline vs 6 weeks: 0.001*
	6 weeks	10 (50%)	10 (50%)		Baseline vs 3 months: <0.001*
	3 months	9 (45%)	11 (55%)		6 weeks vs 3 months: 1.000

**Table 5:** Intergroup comparison of bleeding on probing *A significant difference at p ≤ 0.05 (statistically significant) SD, standard deviation

Interval	Group I (test)		Group II (control)		p-value
	Yes	No	Mean	SD	
Baseline	20 (100%)	0	20 (100%)	0	--
6 weeks	3 (15%)	17 (85%)	10 (50%)	10 (50%)	0.041*
3 months	0	20 (100%)	9 (45%)	11 (55%)	0.001*

## Discussion

Periodontal inflammation of the tooth’s supporting tissues is a progressively destructive change that causes bone and ligament loss. Effective periodontal therapy for plaque-related diseases requires eliminating inflammation by restoring a clean root surface that is biologically acceptable. All existing therapeutic methods strive to accomplish this while preserving as much cementum as possible. This core, potentially age-old mechanical approach to therapy can be supplemented with systemic antibiotics, local antibacterial agents, and host-modulating agents.

The periodontal pocket is a crucial site for delivering antibacterial drugs, allowing for the establishment and maintenance of local concentrations directly at the disease site for the desired duration. Administering just a few milligrams of an antibacterial or anti-inflammatory agent via controlled local delivery within the periodontal pocket can sustain therapeutic levels in the crevicular fluid longer than any other delivery method. Consequently, developing improved strategies for precisely administering medications into the periodontal pocket is essential, as it minimizes undesirable effects like systemic antibiotic absorption and the development of bacterial resistance.

Biological intervention in periodontics is rapidly advancing, with the emergence of more recent techniques like bone morphogenic proteins and platelet-rich concentrates commonly used to facilitate the healing process and mitigate inflammation. The placenta, being a biological tissue, acts as an innate repository for many biologically active constituents with potent healing properties. Although evidence of placental extracts being used as a wound healing agent in oral conditions supports their regenerative, anti-inflammatory, and antioxidative properties, there is currently insufficient literature to justify the use of human placental extracts as a local drug delivery agent in patients with chronic periodontitis [8,11].

Therefore, the present study aimed to evaluate changes in clinical soft tissue parameters with SRP with and without placental extract gel as a local drug delivery under magnification to investigate the therapeutic benefits of placental extracts and their role in the treatment of chronic periodontitis.

The placental extract gel was adsorbed in a collagen plug (Fix Plug™, Synerheal) to increase its substantivity within the pocket. Typically, the collagen plug was cut into very small particles and mixed with gel until its complete adsorption. The collagen plug containing human placental extract gel was firmly inserted into the pocket, where it quickly became fixed. The collagen plug serves as a carrier, as it can increase the substantivity of the local drug delivery by up to 10-28 days [14-16]. The present study incorporated magnification as a vital component of the methodology. Magnification in dental procedures has gained recognition for its ability to enhance precision, visualization, and overall procedural success [12,17]. It allowed a detailed and magnified view of the treatment sites, enabling operators to visualize and access areas with greater precision. This enhanced visibility is crucial, especially in periodontal procedures where accurate assessment and treatment at the subgingival level are paramount. It also facilitated a thorough removal of calculus and plaque, ensuring a more effective treatment outcome. The ability to visualize and address intricate details at a magnified level contributed to the success of the intervention. The magnification used in the study minimized the likelihood of procedural errors by providing a clear and enlarged view of the treatment field. This reduction in errors is crucial in ensuring the reliability of the study results and the validity of the comparisons between the test and control groups. This aligns with contemporary dental practices that emphasize technological integration for enhanced clinical efficacy. 

In the present study, group I exhibited significantly lower GI scores at six weeks and three months compared to group II, indicating better gingival health in the test group. However, there were no statistically significant variations in PI scores between the two groups at any time point, indicating that the two treatment modalities produced similar plaque control outcomes. Group I also demonstrated significantly lower PPD at six weeks and three months compared to group II, indicating greater reductions in PPD with the adjunctive use of placental extract gel. While RAL did not differ significantly between the two groups at any time point, the test group consistently outperformed the control group in terms of BoP outcomes.

The improvement in clinical parameters in the present study is in corroboration with a study done by Yoshida et al. [18]. The researchers conducted a clinical evaluation to assess the administration of Placenta Lucchini in periodontal diseases. The treatment resulted in a reduction in BoP, indicating a decrease in inflammation. There was also a decrease in PPD and clinical attachment gain, which is associated with improved periodontal health. Other studies by Sharma et al. [19] and Calvarano et al. [20] showed improved periodontal health with the use of placental extracts. Morsy et al. [21] analyzed the healing of epithelium, connective tissue, and alveolar bone in rats subjected to experimental periodontitis with SRP alone and SRP with human placental extracts (placentrex). She concluded that the placental extract group could be used as a local drug delivery system in combination with SRP for the treatment of periodontitis due to its potential to decrease inflammation by decreasing the inflammatory cell mass and TNF-α immune expression. It also increases vascularization, enhances the formation of alveolar bone, and improves the condition of periodontal ligament fibers.

In vitro studies evaluated the effect of placental extracts on human gingival fibroblasts. There was enhanced production of type I collagen in HGF placental extracts directly related to the regenerative capabilities of periodontal tissues [22,23].

The findings of this study suggest that the incorporation of placental extract gel as a local drug delivery agent in conjunction with SRP can result in enhanced periodontal treatment outcomes. The improvements in gingival health, plaque control, PPD reduction, and BoP incidence highlight the potential clinical benefits of this adjunctive therapy.

Despite the positive outcomes, one notable limitation of the present study is that it only addresses chronic localized periodontitis, and the results may not be directly applicable to other types of periodontal diseases or generalized periodontitis. Furthermore, the study was conducted at a single center, which limits the external validity and generalizability of the findings. Hence, more research with a diverse population is needed to determine the benefits and drawbacks of using human placental extracts in the treatment of periodontal diseases.

## Conclusions

In conclusion, this study has sought to contribute valuable insights into the treatment landscape of chronic localized periodontitis by comparing the efficacy of SRP with and without the incorporation of placental extract gel, all conducted under magnification. The study demonstrated that adjunctive treatment with placental extract gel alongside SRP led to significant improvements in gingival health and reduced pocket depth compared to SRP alone. These findings suggest the potential efficacy of placental extract gel as a local drug delivery agent in the management of chronic localized periodontitis. The amalgamation of conventional techniques with innovative approaches, such as placental extract gel, opens avenues for more tailored and efficacious periodontal therapies. As we conclude this study, we anticipate that our findings will catalyze further investigations, ultimately benefiting both clinicians and patients in the pursuit of enhanced periodontal care.

## References

[1] Armitage GC (2004). Periodontal diagnoses and classification of periodontal diseases. Periodontol 2000.

[2] Krishna R, De Stefano JA (2016). Ultrasonic vs. hand instrumentation in periodontal therapy: clinical outcomes. Periodontol 2000.

[3] Sbordone L, Ramaglia L, Gulletta E, Iacono V (1990). Recolonization of the subgingival microflora after scaling and root planing in human periodontitis. J Periodontol.

[4] Ali RW, Lie T, Skaug N (1992). Early effects of periodontal therapy on the detection frequency of four putative periodontal pathogens in adults. J Periodontol.

[5] Dzink JL, Gibbons RJ, Childs WC 3rd, Socransky SS (1989). The predominant cultivable microbiota of crevicular epithelial cells. Oral Microbiol Immunol.

[6] Jorgensen MG, Slots J (2000). Responsible use of antimicrobials in periodontics. J Calif Dent Assoc.

[7] Tonello G, Daglio M, Zaccarelli N, Sottofattori E, Mazzei M, Balbi A (1996). Characterization and quantitation of the active polynucleotide fraction (PDRN) from human placenta, a tissue repair stimulating agent. J Pharm Biomed Anal.

[8] Gwam C, Ohanele C, Hamby J, Chughtai N, Mufti Z, Ma X (2023). Human placental extract: a potential therapeutic in treating osteoarthritis. Ann Transl Med.

[9] Hong JW, Lee WJ, Hahn SB, Kim BJ, Lew DH (2010). The effect of human placenta extract in a wound healing model. Ann Plast Surg.

[10] Chakraborty PD, Bhattacharyya D, Pal S, Ali N (2006). In vitro induction of nitric oxide by mouse peritoneal macrophages treated with human placental extract. Int Immunopharmacol.

[11] Chakraborty PD, Bhattacharyya D, Pal S, Ali N (2005). In vitro growth inhibition of microbes by human placental extract. Current Science.

[12] Hoerler SB, Branson BG, High AM, Mitchell TV (2012). Effects of dental magnification lenses on indirect vision: a pilot study. J Dent Hyg.

[13] Löe H (1967). The gingival index, the plaque index, and the retention index systems. J Periodontol.

[14] Minabe M, Uematsu A, Nishijima K (1989). Application of a local drug delivery system to periodontal therapy: I. Development of collagen preparations with immobilized tetracycline. J Periodontol.

[15] Mehta S, Humphrey JS, Schenkman DI, Seaber AV, Vail TP (1996). Gentamicin distribution from a collagen carrier. J Orthop Res.

[16] Choi J, Park H, Kim T (2014). Engineered collagen hydrogels for the sustained release of biomolecules and imaging agents: promoting the growth of human gingival cells. Int J Nanomedicine.

[17] Dadwal A, Kaur R, Jindal V, Jain A, Mahajan A, Goel A (2018). Comparative evaluation of manual scaling and root planing with or without magnification loupes using scanning electron microscope: a pilot study. J Indian Soc Periodontol.

[18] Yoshida M, Yamamoto H, Muraoka T, Otsu S, Tsuruta M, Yamamoto M, Toyota M (1967). A clinical evaluation of placenta Lucchini administration in periodontal diseases [I]. J Kyushu Dent Soc.

[19] Sharma A, Sharma S, Nagar A (2020). Comparative evaluation to assess the effect of SRP with or without human placental extracts as local drug delivery in treatment of localized periodontal pocket-a randomized controlled clinical trial. Int J Dent Res.

[20] Calvarano G, De Polis F, Sabatini G (1989). Treatment with placental extract in periodontal disease. Dent Cadmos.

[21] Morsy SM, El-Sherbiny RH, Shata MS (2022). Assessment of the effect of local administration of placental extract gel on the treatment of experimental periodontitis in rats: a histopathologic and immunohistochemical study. Egypt Dent J.

[22] Honda Y, Imamura Y, Fukui T, Masuno K, Wang P (2015). Effect of placenta on collagen type-1 and inflammatory cytokine production in human gingival fibroblasts. Oral Ther Pharm.

[23] Akagi H, Imamura Y, Makita Y, Nakamura H, Hasegawa N, Fujiwara S, Wang P L (2016). Evaluation of collagen type-1 production and anti-inflammatory activities of human placental extracts in human gingival fibroblasts. J Hard Tissue Biol.

